# Antimicrobial Use and Prescribing Patterns in Three Nepalese Hospitals: Findings from the 2022 Global-PPS

**DOI:** 10.3390/antibiotics15090936

**Published:** 2026-09-20

**Authors:** Anup Luitel, Roshani Salami Magar, Gita Paudel, Beena Jha, Aruna Luitel, Samyukta Khatri Chettri, Rajan Shrestha, Lalit Mohan Pant, Maxencia Nabiryo, Bridget Kebirungi, Agbaje Ganiyu Olawale, Samantha Weston, Frances Garraghan, Diane Ashiru-Oredope, Victoria Rutter, Kelly Alexander

**Affiliations:** 1 Department of Pharmacy, School of Science, Kathmandu University, JG9Q+PGG, Dhulikhel 45200, Nepal; rm45011719@student.ku.edu.np (R.S.M.); rajanshrestha@ku.edu.np (R.S.); lalit.pant@ku.edu.np (L.M.P.); 2 Department of Pharmacology, Chitwan Medical College, Bharatpur 44200, Nepal; 3 Department of Microbiology, Kathmandu Medical College, Baburam Acharya Road, Kathmandu 44600, Nepal; 4 Aarogya Multispeciality Hospital, M9CH+G47, Sundarnagar, Madhyapur Thimi 44600, Nepal; 5 National Academy of Medical Sciences, Surya Bikram Gyawali Marg, Kathmandu 44600, Nepal; kcsamyukta@gmail.com; 6 Commonwealth Pharmacists Association, Royal Pharmaceutical Society, 66–68 East Smithfield, London E1W 1AW, UKdiane.ashiru-oredope@commonwealthpharmacy.org (D.A.-O.);; 7 One Health, College of Medicine and Veterinary Medicine, the Royal (Dick) School of Veterinary Studies, University of Edinburgh, Old College, South Bridge, Edinburgh EH8 9YL, UK; 8 School of Health & Life Sciences, Teesside University, Middlesbrough TS1 3BX, UK; 9 British Society for Antimicrobial Chemotherapy (BSAC), 53 Regen Pl, Birmingham B1 3NJ, UK; 10 School of Pharmacy, University of Nottingham, Nottingham NG7 2RD, UK

**Keywords:** antimicrobial resistance, antimicrobial prescribing, Global-PPS, antimicrobial stewardship, Nepal, tertiary hospitals

## Abstract

**Background/Objectives**: Antimicrobial resistance (AMR) poses a global threat, exacerbated by the overuse and misuse of antimicrobials. This study evaluated antimicrobial use and prescribing patterns among inpatients in three tertiary hospitals in Nepal. **Methods**: A cross-sectional point prevalence survey of the inpatient module of the standardized Global-PPS methodology was conducted across three tertiary hospitals in Nepal during the 2022 Global-PPS Period 2 (P2) survey, carried out between May and August 2022. Data were collected via a paper-based form (University of Antwerp;) and analyzed descriptively. Antimicrobial use was defined as the proportion of inpatients receiving at least one systemic antimicrobial on the day of the survey. **Results**: Of 379 patients eligible for the study, 233 (61.5%) received at least one antimicrobial, with the highest prevalence observed in adult and neonatal intensive care units (ICUs). Out of 371 antibiotic prescriptions, third-generation cephalosporins (30.2%) and carbapenems (8.1%) were the most frequently prescribed, and 61.0% of overall antibiotics belonged to the Watch group of WHO AWaRe classification. Surgical prophylaxis often exceeded recommended durations, with 91.5% of prophylactic antibiotics prescribed for more than one day. Empirical therapy dominated 56.33% of all prescriptions, with targeted therapy limited to 4.04%. **Conclusions**: The high overall prevalence of antimicrobial use, the reliance on empirical therapy, and over-reliance on Watch-group antibiotics and Unclassified AWaRe antibiotics highlights the urgent need for robust antimicrobial stewardship programs in Nepal.

## 1. Introduction

Antimicrobial resistance (AMR) is a major global health problem because it makes it harder to prevent and treat infections and slows progress in modern medicine. As more bacteria become resistant, hospital stay gets longer, treatment failure increases, and more people die worldwide. The Lancet estimated that bacterial AMR directly caused about 1.27 million deaths and was linked to nearly 4.95 million deaths globally. The impact is especially severe in low- and middle-income countries (LMICs), where health systems often lack good diagnostics, effective surveillance, and enough resources for antimicrobial stewardship (AMS) [1,2].

A major contributor to the development of antimicrobial resistance (AMR) is the misuse of antimicrobials in people, animals, and agriculture. Examples of antibiotic misuse include unnecessary antibiotic prescriptions, overuse of broad-spectrum antibiotics, long treatment courses, self-medication, and not following treatment guidelines. All these contribute to the development and prevalence of resistant bacteria in healthcare settings [1,2]. Other issues, such as poor infection control practices and inadequate sanitation, contribute to the spread of resistant infections in these settings. In addition, weak antibiotic policies, and limited access to diagnostic services, especially in low-resource areas, also make the challenge of treating infection and managing AMR worse [2,3]. To address this, the World Health Organization (WHO) has made antimicrobial stewardship (AMS) a priority in its Global Action Plan on AMR and introduced the Access, Watch, and Reserve (AWaRe) classification to promote better prescribing. The WHO recommends that Access antibiotics should account for at least 60% of national antibiotic use [2,4].

Nepal, a low- and middle-income country in South Asia, faces several challenges with antimicrobial stewardship (AMS). These include insufficient trained staff, frequent use of antibiotics without lab confirmation, and low awareness of AMS among healthcare workers [5]. The Nepal National Action Plan on AMR (2021–2026, now replaced by the Second NAP-AMR 2024–2028) aims to improve surveillance using a One Health approach. Nepal also reports data to the Antimicrobial Resistance and antimicrobial consumption (AMC) sections of the WHO GLASS system. According to the latest WHO report, only 26.24% of antibiotics used in Nepal in 2023 were from the WHO AWaRe Access group, showing a heavy reliance on Watch-group antibiotics [6]. Data from 26 hospitals also show high rates of multidrug resistance in common bacteria including *Escherichia coli* (51%), *Klebsiella* spp. (56%), and *Acinetobacter* spp. (72%) [7]. However, regular and standardized monitoring of antibiotic use in hospitals is still rare, and most evidence comes from a few single-center studies.

Although some studies reporting point prevalence surveys (PPS) in Nepal exist, they are limited in scope. For example, a PPS in six Kathmandu Valley hospitals found ceftriaxone was the most commonly used empirical antibiotic, but did not report on AWaRe classification or quality indicators by hospital ward [8]. Another PPS focused on ICUs found high rates of antibiotic prescribing and empirical treatment, but only studied one ward in one hospital, so it could not compare different hospital settings [9]. Neither study used a standardized, validated tool across several tertiary hospitals or looked at key prescribing quality indicators like guideline adherence, documentation of treatment reasons, or duration of antibiotics for surgical prophylaxis. These indicators are important for setting specific stewardship improvement targets.

To address this gap, this study uses the standardized Global-PPS tool in three tertiary hospitals. Two are large teaching hospitals that provide both undergraduate and postgraduate medical education and specialist care. The third is a private multispecialty referral hospital serving mainly urban and peri-urban populations. Because these hospitals receive referrals from primary and secondary healthcare facilities across Bagmati Province and nearby areas, the study can compare different wards, including adult and neonatal intensive care units, as well as pediatric, medical, and surgical wards. This setting is more diverse than earlier single-site studies in Nepal. Unlike previous research, this study reports antibiotic use by WHO AWaRe classification and includes prescribing quality indicators such as guideline adherence, use of empirical versus targeted therapy, and duration of surgical prophylaxis. This provides the first multi-site baseline for prescribing quality in Nepal.

This study aimed to assess antimicrobial use and prescribing practices in three tertiary hospitals in Nepal using the standardized Global-PPS method. Specifically, it compared how often antimicrobials were prescribed in different hospital wards, described antibiotic use by indication and by WHO AWaRe classification, and evaluated prescribing quality indicators such as guideline adherence and duration of antibiotics for surgical prophylaxis. The goal was to identify areas where future stewardship interventions may be needed.

## 2. Results

### 2.1. Antimicrobial Prevalence and Prescription

A total of 379 inpatients were present across the three participating hospital sites during the survey period. Of these, 233 (61.5%) received at least one antimicrobial on the day of Global-PPS. Pooled results show the highest levels of antimicrobial prescribing prevalence were recorded in neonatal intensive care units (95.7%), adult intensive care units (93.2%), and the pediatric medical ward (86.7%). The overall antimicrobial prescribing prevalence by ward type is presented in Table 1.

Among 153 prophylactic antimicrobial prescriptions, surgical prophylaxis accounted for 94 prescriptions (61.4% of prophylactic antimicrobial use), with 91.5% (86 out of 94 prescriptions) frequently extending beyond the recommended 24 h window, while medical prophylaxis accounted for 59 prescriptions (38.6%) (Table 2).

Among 224 therapeutic antimicrobial prescriptions, empirical prescribing was extremely high for the treatment of both hospital-acquired infections (HAI; N = 29, 100%) and community-acquired infections (CAIs; N = 180, 92.3%). In contrast, targeted therapy was infrequently prescribed for CAI (7.7%, N = 15) and was not prescribed for any HAI (0%, N = 0) (Table 2).

The most common route of administration of antimicrobials was parenteral (86.1%). Multiple antibiotic therapy (48.8%) and multiple antibiotic diagnoses were common in the survey (Table 2).

Systemic antibacterials (Anatomical Therapeutic Chemical (ATC) code J01; antibiotics) were the most commonly prescribed class of antimicrobial to 231 (60.9%) of the 379 hospitalized patients. Other antimicrobial classes were less prescribed and included nitroimidazole derivatives (N = 7, 1.8%), antifungal agents (N = 2, 0.5%), anti-tuberculosis drugs (ATBs; N = 1, 0.3%), and intestinal anti-infectives (antimicrobial agents used to treat gastrointestinal infections, ATC subgroup A07A; N = 1, 0.3%). No antiviral or antiparasitic agents were prescribed during the survey period (Table 3).

**Figure 1:**
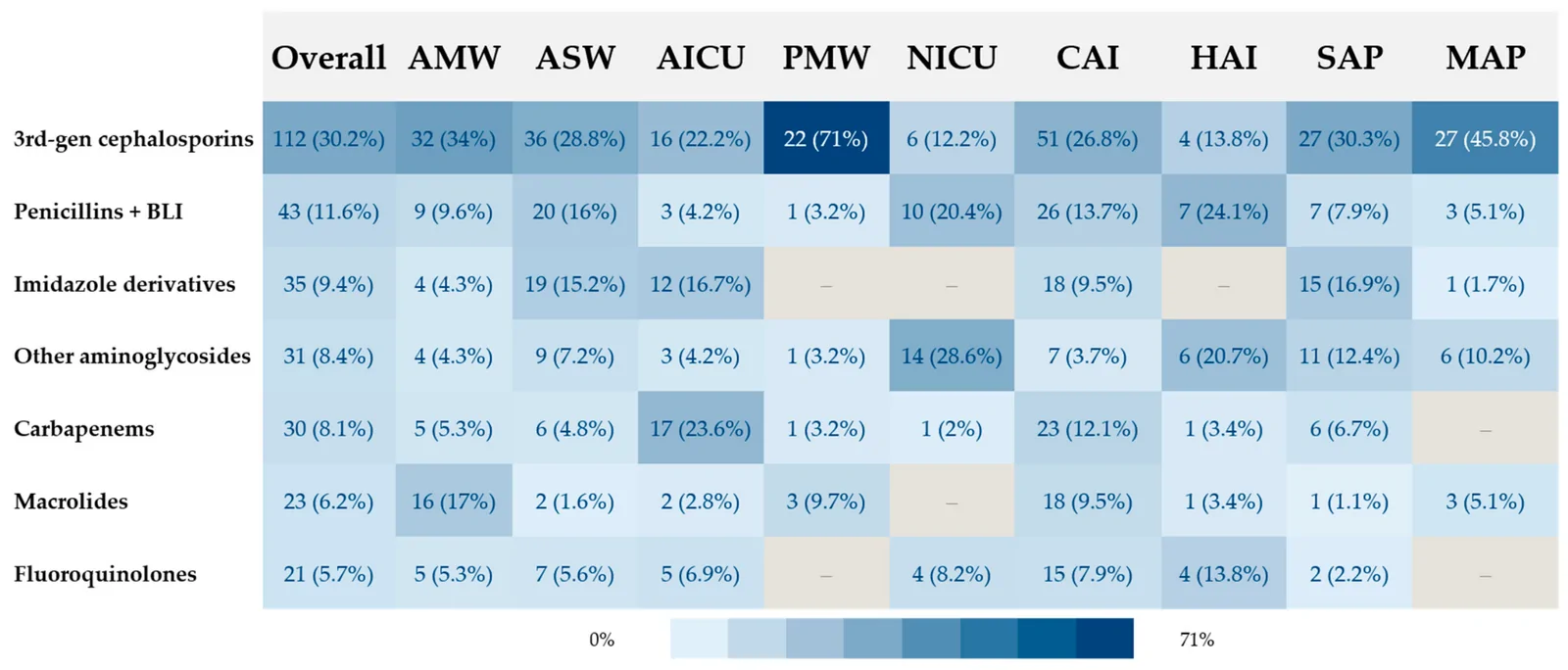
Top prescribed antimicrobial classes by ward and prescribing category, Nepal hospital, Global-PPS 2022–P2. Each cell shows the number of prescriptions (N) and, in parentheses, the percentage that the antimicrobial class represents of all systemic antibacterial (ATC J01) prescriptions within that column’s category. Cell shading is scaled to percentage (darker = higher proportion); a dash indicates the class was not prescribed in that category. Abbreviations: AMW = adult medical ward (N = 94); ASW = adult surgical ward (N = 125); AICU = adult intensive care unit (N = 72); PMW = pediatric medical ward (N = 31); NICU = neonatal intensive care unit (N = 49); CAIs = community-acquired infections (N = 190); HAIs = hospital-acquired infections (N = 29); SAP = surgical antimicrobial prophylaxis (N = 89); MAP = medical antimicrobial prophylaxis (N = 59); overall (N = 371); BLI = beta-lactamase inhibitors; ATC = Anatomical Therapeutic Chemical classification system.

Overall, 371 systemic antibacterial (ATC J01) prescriptions were recorded; the most prescribed antibiotics were third-generation cephalosporins (30.2%, N = 112) of all antibiotic prescriptions, followed by penicillin combined with β-lactamase inhibitors (11.6%, N = 43), imidazole derivatives (9.4%, N = 35), aminoglycosides (8.4%, N = 31), carbapenems (8.1%, N = 30), macrolides (6.2%, N = 23), and fluoroquinolones (5.7%, N = 21). The use of third-generation cephalosporin use was high on the pediatric medical ward (71.0%, N = 22), followed by the adult medical ward (34.0%, N = 32), adult surgical ward (28.8%, N = 36), adult intensive care unit (22.2%, N = 16), and neonatal intensive care unit (12.2%, N = 6). Use of third generation cephalosporins was more frequent in medical antimicrobial prophylaxis (45.8%, N = 27) whereas penicillin combined with β-lactamase inhibitors was more frequent in the treatment of HAIs (24.1%, N = 7). Carbapenems were most frequently prescribed in the adult intensive care unit (23.6%, N = 17), whereas other aminoglycosides were commonly prescribed in the neonatal intensive care unit (28.6%, N = 14). The prescribed antimicrobial classes by ward and prescribing category are presented in Figure 1.

Table 4 shows the most frequently reported indications for antimicrobial use alongside the three most commonly prescribed antimicrobials for each indication. The most common indications for antibiotic prescription were hospital-acquired pneumonia (20.5%), lower urinary tract infection (UTI) treatment (13.1%), and UTI prophylaxis (9.8%). The least prevalent indications were bronchitis (4.1%) and intra-abdominal sepsis (4.1%). The three antimicrobials most commonly prescribed antibiotics for pneumonia were meropenem (J01DH02) (21.5%), ceftriaxone (J01DD04) (19.7%), and azithromycin (J01FA10) (17%). For lower and upper UTIs, levofloxacin (J01MA12) (19.5%), ceftriaxone (17.1%), and piperacillin/tazobactam (17.5%) were most commonly prescribed. For surgical and medical UTI prophylaxis, Imipenem/cilastatin (J01DH51) (69.0%), piperacillin/tazobactam (J01CR05) (37.5%), and doxycycline (J01AA02) (100%) were prescribed.

### 2.2. Antimicrobial Quality Indicators

It is recognized best practice to document the indication for prescribed antimicrobials at the point of initiation. Across all patient groups, the indication was most frequently recorded in the medical notes among ICU (88.7%, N = 110) and surgical patients (87.5%, N = 112), with less frequent documentation in medical wards (61.3%, N = 73). Table 5 provides a breakdown of quality indicators by patient group. Guideline compliance with local antimicrobial treatment guidelines was 28.0% (N = 7) in ICUs, 61.4% (N = 35) in medical wards, and 73.5% (N = 61) in surgical wards (among the adult and neonate patients). Furthermore, local antimicrobial guidelines were frequently not available in ICUs (65.3%, N = 81) and medical wards (37%, N = 44). Documentation of antimicrobial stop or review dates were most frequently documented in ICUs (84.7%, N = 105), with lower rates being observed in medical (52.9%, N = 63) and surgical (46.1%, N = 59) ward settings. A detailed summary of antimicrobial prescribing quality indicators in participating hospital sites by ward is provided in Table 5.

### 2.3. Prescribing of WHO AwaRe Antibiotics

**Figure 2:**
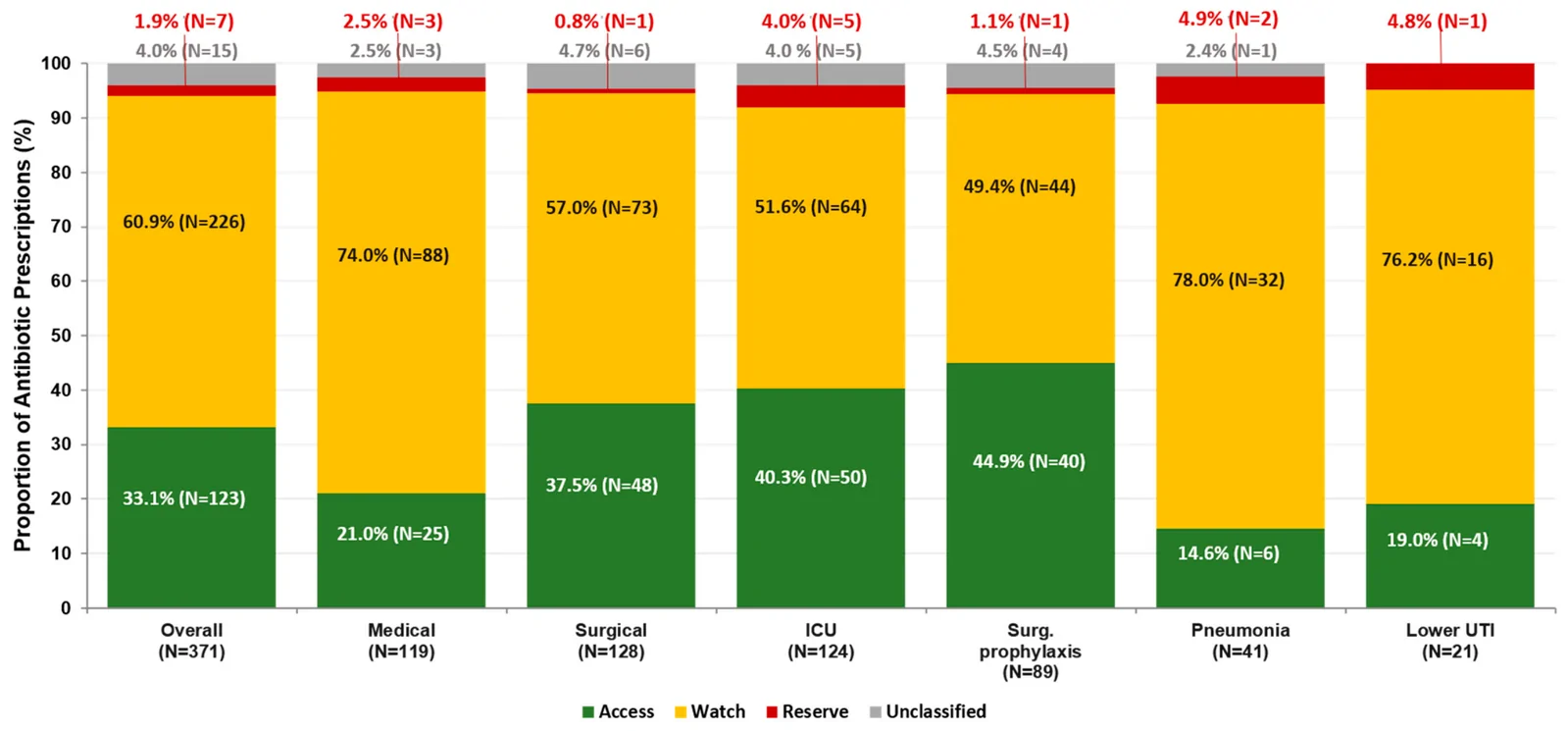
Distribution of antibiotic prescriptions by WHO AWaRe classification across clinical settings and indications in Nepal Hospital, Global-PPS 2022–P2 (N (%) of antibiotic prescriptions by AWaRe category). Note: Percentages for each category are calculated as N/total prescriptions for that clinical setting or indication and are rounded to one decimal place; column totals may therefore differ marginally from 100% due to rounding.

**Figure 3:**
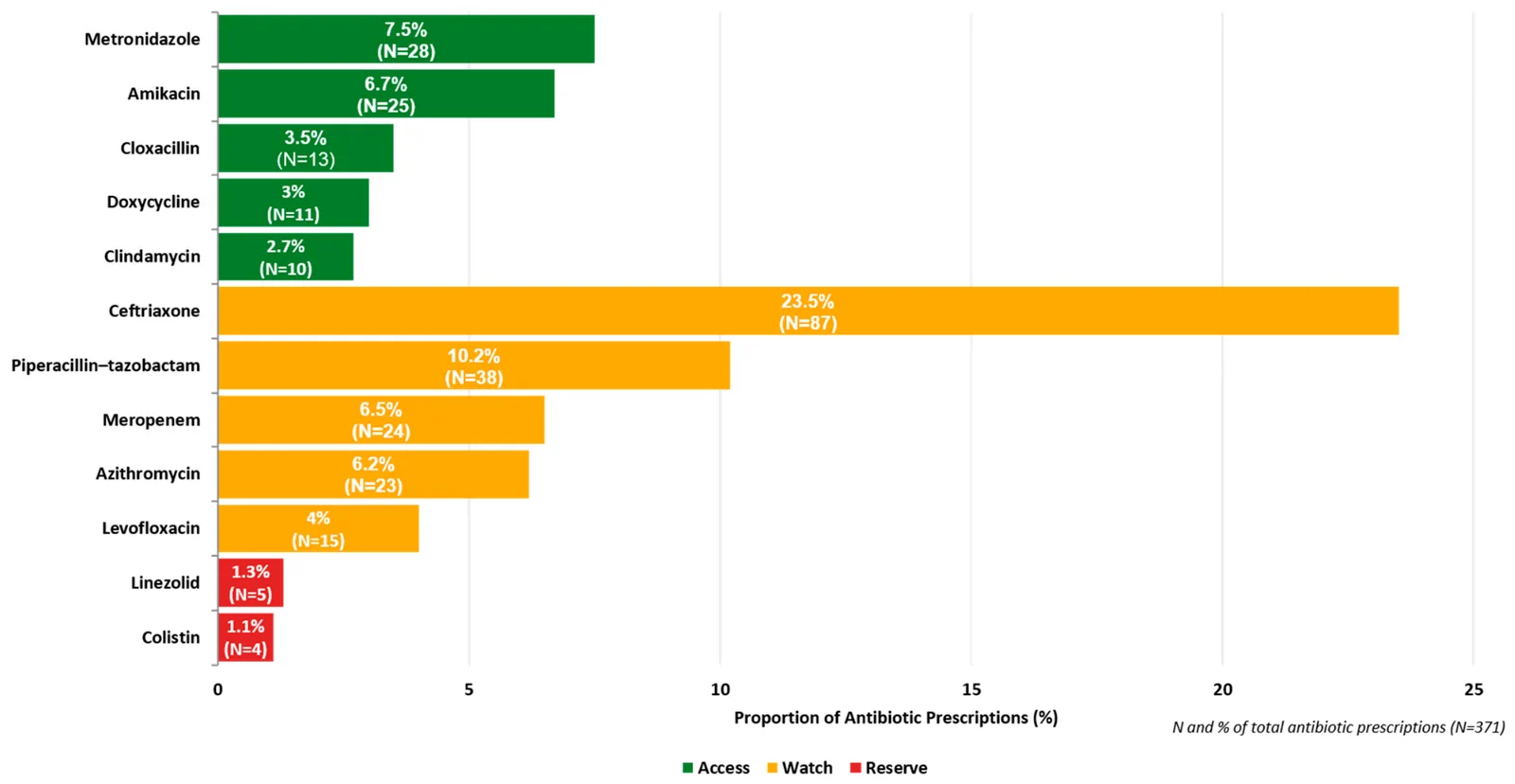
Most frequently prescribed antibiotics by WHO AWaRe category in Nepal Hospital, Global-PPS 2022–P2 (Top agents per category, % of total antibiotic prescriptions, N = 371).

Antibiotics within the Watch group accounted for 61.0% of the total 371 prescriptions in the survey, followed by the Access group (33.1%), Reserve (1.9%), and Unclassified (4.0%). The medical departments had the highest prescribing rate of Watch-group antibiotics (74.0%), followed by surgical departments (57.0%) and ICUs (51.6%). The most frequently prescribed antibiotics were ceftriaxone (23.5%) and piperacillin–tazobactam (10.2%). Reserve antibiotics, Linezolid and Colistin, were used infrequently. WHO AWaRe “Unclassified” antimicrobial use was more prevalent in lower urinary tract infections (5.0%), surgical prophylaxis (4.0%), and hospital-acquired pneumonia (2.0%). The distribution of antibiotic prescriptions according to WHO AWaRe Classification in the survey are presented in Figure 2 and Figure 3.

## 3. Discussion

This multicenter Global-PPS study describes antimicrobial prescribing patterns, prescribing quality indicators, and WHO AWaRe classification across three private tertiary-care hospitals: two in Kathmandu and one in Bharatpur, Nepal. Overall antimicrobial prevalence was 61.5%, empirical therapy accounted for the large majority of therapeutic prescribing (92.3% of community-acquired infections, 100% of hospital-acquired infections), surgical prophylaxis frequently lasted beyond the recommended 24 h window (91.5% of cases), and Watch-group antibiotics comprised 61% of prescriptions.

Overall prevalence of antimicrobial prescribing (61.5%) was comparable to South Asia (62.6%) [10], higher than Tanzania (38%), and lower than Nigeria (69.7%) [11,12], and was higher than China (56%) [13] and Saudi Arabia (46.9%) [14]. Such cross-country variation probably reflects differences in infectious disease burden, diagnostic capacity, and AMS implementation, although this cross-sectional design cannot establish which factors are responsible. Third-generation cephalosporins accounted for 30.2% of prescriptions overall and rose to 71.0% in paediatric medical wards, in line with reports from India and Bangladesh, where use has reached up to 68% [15]. Ceftriaxone alone was the most prescribed agent (23.5%), matching a prior Kathmandu report (26.7%) [8] and comparable to figures from Scotland (27.9%) and Pakistan (31.4%) [16,17].

Empirical prescribing was the main approach for treatment, making up 92.3% of community-acquired infections and 100% of hospital-acquired infections. This is similar to Rwanda, where slow culture results and limited lab capacity led to high empirical use (76.5%) [18]. Similar challenges likely explain the low rate of targeted therapy here (7.7%). In contrast, targeted therapy in the United States increased from 35% to 45% between 2011 and 2015 as diagnostics improved [19]. High empirical use is not always inappropriate; for many serious infections, starting treatment quickly while waiting for cultures is recommended. The bigger concern is the lack of follow-up to adjust therapy, since this survey cannot tell if cultures were never done, were negative, or did not lead to changes in treatment. Improving lab capacity, encouraging routine culture reviews, and adding structured antimicrobial reassessment at 48–72 h would help identify where the real gaps are, while also strengthening the quality of antimicrobial resistance surveillance data that informs future empirical therapy choices.

Surgical prophylaxis accounted for 61.4% of prophylactic use, and 91.5% of these prescriptions lasted more than 24 h. This is similar to findings from Ghana and Vietnam (59.1%) [15,20] and is a clear example of inappropriate prescribing, since international guidelines recommend single-dose or 24 h surgical prophylaxis for most procedures. Introducing standardized preoperative protocols, regular audits of prophylaxis duration, and prescriber education would be practical steps to address this issue.

Not every indication-specific pattern can be judged with equal confidence. Not all patterns for specific indications can be judged with certainty. Meropenem (21.5%), ceftriaxone (19.7%), and azithromycin (17.0%) were most commonly used for hospital-acquired pneumonia, while levofloxacin (19.5%), piperacillin/tazobactam (17.5%), and ceftriaxone (17.1%) were most commonly used for lower urinary tract infections. Using broad-spectrum antibiotics in these cases may be justified by severity, previous antibiotic use, or local resistance, but this survey did not collect the microbiological or clinical severity data needed to confirm this. The high use of a carbapenem (imipenem/cilastatin, 69.0%) for UTI surgical prophylaxis is more difficult to justify, as stewardship guidelines usually reserve carbapenems for confirmed or likely multidrug-resistant infections, not routine prophylaxis. This finding should be reviewed through direct clinical audit. The exclusive use of doxycycline (100%) for UTI medical prophylaxis was based on a small number of prescriptions and cannot be judged as appropriate or not. Similar use of broad-spectrum agents has been reported in Bangladesh [21], Pakistan [19], Ghana [15], Tanzania [12], Kenya [22], Nigeria [11], and Rwanda [18], often due to limited diagnostics and high AMR burden. Addressing this will likely require more culture-guided therapy, better adherence to evidence-based guidelines, and regular prescription audits with feedback [2,3,23]. Guideline compliance in ICUs (28.0%) was low, compared with medical (61.4%) and surgical (73.5%) wards, and guidelines were simply unavailable for 65.3% of ICU indications and 37.0% of medical ward indications. These values fall well below the compliance rates above 80% typically reported in high-income settings [23,24]. Pediatric prescribing showed better guideline adherence (over 70%) than adult prescribing (over 50%), a pattern also seen in China (75%) [13] and Ghana [15], and possibly reflecting more developed pediatric-specific AMS protocols. This study did not directly test that explanation, so it is still speculative.

According to the WHO AWaRe classification, Watch-group antibiotics accounted for 61% of prescriptions, similar to rates in Rwanda (65%) and Bangladesh (64%) [18,21], and higher than the WHO benchmark for country-level use, which recommends at least 60% Access-group use [4]. This means Access-group antibiotic use was below the recommended level; however, noting that specific hospital-level targets have not been set. The remaining 4% of prescriptions were Unclassified, meaning they had not yet been assigned an Access, Watch, Reserve, or Not Recommended category in the AWaRe index at the time of analysis, often because the specific agent or fixed-dose combination prescribed was not yet listed. No prescriptions in this survey fell into the WHO “Not Recommended” category itself. While Unclassified prescriptions are not necessarily inappropriate, their absence from the AWaRe framework limits stewardship monitoring and benchmarking against WHO targets, and hospitals should periodically cross-check locally used antimicrobials against the current AWaRe index to keep classification current. It is important to remember that WHO’s AWaRe targets are for overall antibiotic use, including outpatients; hospital inpatients, especially in intensive care and surgical wards, are expected to use more Watch-group antibiotics than the general population. So, the 60% Access target is a general guideline, not a strict ward-level rule. The use of multiple antimicrobials, seen in 48.8% of patients, also increases the risk of resistance development; local, ward-specific prescribing guidelines are essential, particularly for intensive care units and surgical prophylaxis. Formal protocols should restrict surgical prophylaxis to 24 h or less. Implementation of culture-before-antibiotic policies, combined with structured review at 48–72 h, is recommended. Prospective audit and feedback should focus on the most frequently used agents (third-generation cephalosporins, carbapenems) and indications (urinary tract infection and pneumonia prophylaxis and treatment). Regular repetition of Global-PPS surveys would facilitate monitoring of progress, while ward-level dashboards summarizing prescribing and AWaRe metrics, together with structured prescriber education, could enhance daily clinical decision making [25]. In contexts where routine microbiological surveillance remains limited, digital and predictive decision-support tools may provide valuable support. A recent meta-analysis demonstrated that artificial intelligence and machine-learning models outperformed traditional risk-scoring systems in sensitivity and negative predictive value for stewardship decisions, although external validation is limited and issues regarding explainability and regulation must be addressed before widespread clinical adoption [26].

This study has several limitations. Data were collected from three private tertiary-care hospitals—two in Kathmandu Valley and one in Bharatpur—so the results may not apply to public-sector, rural, or lower-level facilities. A prior six-hospital point prevalence survey in the Kathmandu Valley also included private hospitals [8], so private-sector setting alone is not a novel feature of this study; rather, this study extends that evidence base by adding ward-level AWaRe classification and prescribing-quality indicators (guideline compliance, indication documentation, and stop/review dates), which were not reported in the earlier survey. Because this was a single time-point survey, it cannot show seasonal changes, trends over time, or whether the patterns found here are stable or just a snapshot. Demographic data like age, sex, and comorbidities were not collected, so we could not see if prescribing varied by patient group or case mix. Finally, as with all point prevalence surveys, this study recorded prescribed, not confirmed administered, antimicrobials. We did not check for differences between what was prescribed and what was actually given, such as missed or stopped doses, so the true prevalence of antimicrobial exposure may be slightly overestimated.

Some questions remain. Repeating the PPS over time would help determine if high empirical prescribing and extended surgical prophylaxis are ongoing practices or just temporary changes, and whether the recommended interventions actually improve prescribing. Future surveys that collect demographic and microbiological data would help distinguish clinically justified broad-spectrum use from avoidable stewardship gaps, especially regarding carbapenem use for prophylaxis. Finally, testing digital or AI-supported stewardship tools in resource-limited Nepali hospitals would show if these approaches can realistically improve surveillance where lab and staffing resources are limited.

## 4. Materials and Methods

### 4.1. The Study Design, Setting, and Time Period

A multicenter, cross-sectional point prevalence survey (PPS) of antimicrobial use was conducted among inpatients at three hospitals in Nepal during the 2022 Global-PPS Period 2 (P2) survey, carried out between May and August 2022. The standardized Global-PPS inpatient methodology [27] was used to monitor antimicrobial consumption and evaluate the quality of antimicrobial prescribing practices [18,28]. The Global-PPS is an internationally validated surveillance methodology developed by the University of Antwerp to assess antimicrobial consumption, prescribing quality, and antimicrobial stewardship indicators among hospitalized patients.

### 4.2. Hospital Selection and Setting

Nepal’s Ministry of Health and Population (MoHP) randomly selected participating hospitals. Participation was voluntary; all invited hospitals enrolled and completed the survey, with no declines or attrition. As the specific sampling frame used by MoHP for hospital selection is not available to the study authors, the representativeness of the participating hospitals relative to the larger population of Nepalese hospitals cannot be fully assessed, and findings may not be generalizable to all hospitals in Nepal.

The three hospitals that took part in the study had no previous experience conducting a Global-PPS or any other point prevalence survey of antimicrobial use, and the site champions and data collectors received training in the study procedures and data collection tools before implementation began.

The three participating sites were as follows:

- Two tertiary teaching hospitals were included: one in Kathmandu and the other in Bharatpur, Chitwan, each with 750 inpatient beds. They are large tertiary referral centers which also offer undergraduate and postgraduate medical education as well as comprehensive specialist services.
- One private multispecialty urban hospital in Kathmandu with 50 inpatient beds that mainly caters to urban and peri-urban areas.

Together, these organizations have 1550 inpatient beds and receive referrals from healthcare facilities at primary and secondary levels in Bagmati Province and other parts of Nepal.

### 4.3. The Study Population and Eligibility Criteria

The study included patients admitted to the hospital wards before 08:00 on the day of the survey, in keeping with the Global-PPS method.

Inclusion criteria were that patients were on the ward during the 24 h period before 08:00 on the day of the survey.

Exclusion criteria included day-case admissions and outpatients; patients admitted after 08:00 on the day of the survey; patients discharged before 08:00 on the day of the survey; and cases with incomplete essential information.

The demographic details of the patients, such as age and sex, were not included in this analysis, since the study focused on factors at the prescribing level rather than at the individual patient level.

### 4.4. Data Collection

The data were collected by site champions and data collectors who had been trained in the Global-PPS methodology by a qualified trainer using standard paper-based Global-PPS data collection forms.

The trained personnel assessed the level of antimicrobial exposure by examining each patient’s medical record and medication Kardex (the medication administration record) in order to establish whether or not the patient was receiving at least one systemic antimicrobial on the day of the survey, in accordance with standardized GPPS methodology. Patient interviews were not conducted, and pharmacy dispensing records were not included in the data sources used.

For the purpose of data collection, two forms were employed: a ward form, which recorded the relevant patient history for all patients on the ward at the time of the survey; and a patient form, which collected more detailed information about patients who were receiving antimicrobial treatment, including the antimicrobials prescribed, the route of administration, the indication for therapy (whether it was for a community-acquired infection, a healthcare-associated infection, medical prophylaxis, surgical prophylaxis, or other according to GPPS definitions), the diagnosis, and aspects of prescribing quality such as the review dates, the use of microbiological investigation, and conformity with local guidelines (where these were available). The generic name of each antimicrobial prescribed was noted and coded using the standardized Global-PPS antimicrobial coding system.

Guideline compliance was assessed against each hospital’s own local antimicrobial treatment guidelines (i.e., guidelines developed and adopted by the hospital itself, whether applied hospital-wide or at the ward level), where these existed at the ward level; data collectors recorded, for each antimicrobial prescription, whether a local guideline was available for the relevant indication and, where one was available, whether the prescribed antimicrobial, dose, and duration conformed to it. Wards without an applicable local guideline at the time of the survey were recorded as “guideline not available” rather than non-compliant. Although Nepal’s first National Antibiotic Treatment Guideline was published in 2014 (revised as the National Antimicrobial Treatment Guideline in 2023, after this survey was conducted), no standardized national or international reference guideline was substituted when a local guideline was absent.

The completed paper forms were checked against the eligibility criteria listed above and then input into the Global-PPS web application (University of Antwerp) for validation and analysis.

### 4.5. Antimicrobial Classification

The antimicrobials were grouped using the 2022 version of the World Health Organization Anatomical Therapeutic Chemical (ATC) classification system at ATC level 4 (the chemical subgroup). The analysis of antibiotic use focused on those antibacterials intended for systemic use (those in ATC group J01); non-systemic antimicrobial preparations (such as dermatological, intravaginal, and intestinal formulations) are not included in ATC J01 and have therefore been excluded, in keeping with the scope of the Global-PPS inpatient module, which only includes data on systemic antimicrobial use.

According to the 2023 WHO AWaRe classification [4], antibiotics were divided into Access, Watch, Reserve, or “Not Recommended” groups, the classification being applied by the Global-PPS web application at the time of the data analysis and reporting rather than using the edition that was current when the data were collected in 2022. Methenamine (ATC J01XX05) was included in the analysis whenever it was prescribed, in accordance with its classification as a systemic antibacterial (ATC J01) within the standard Global-PPS inpatient module methodology.

One prescription in the survey was classified under intestinal anti-infectives, a class of antimicrobial agents (ATC subgroup A07A) used to treat gastrointestinal infections; unlike other non-systemic antimicrobial preparations, this agent was captured by the Global-PPS inpatient module because it was prescribed and administered as part of systemic antimicrobial therapy.

### 4.6. Measures of Antimicrobial Use

The prevalence of antimicrobial use was the primary measure of antimicrobial use in this study, defined as the proportion of eligible inpatients who received at least one systemic antimicrobial on the day of the survey:

Prevalence of antimicrobial use (%) = (Number of inpatients receiving ≥1 antimicrobial × 100)/Total number of eligible inpatients surveyed

In addition, we recorded the number of antimicrobial prescriptions for each patient receiving antimicrobial therapy. An antimicrobial prescription was defined as an individual systemic antimicrobial agent prescribed to a patient on the survey day; a patient receiving more than one antimicrobial therefore contributed more than one prescription. The total number and distribution of antimicrobial prescriptions were assessed according to antimicrobial class (ATC), individual agent, WHO AWaRe category, indication, and route of administration.

### 4.7. Statistical Analysis

Descriptive analysis—including prevalence, proportions, and AWaRe classification breakdowns—was generated using the Global-PPS web application (University of Antwerp). Anonymized output was subsequently exported to Microsoft Excel (2021) for additional tabulation and presentation. Results are reported as frequencies and percentages. No inferential statistical tests were performed, consistent with the descriptive objective of the point prevalence survey.

## 5. Conclusions

This multicenter Global-PPS provides important baseline evidence on antimicrobial prescribing patterns across the participating Nepalese hospitals. The findings demonstrate a high prevalence of antimicrobial use, widespread empirical prescribing, predominant use of Watch-group antibiotics, and important gaps in AMS practices, including limited guideline availability and variable adherence across clinical settings.

In a setting where routine antimicrobial use surveillance remains limited, these findings contribute to the national evidence base needed to support the establishment and prioritization of AMS initiatives. Regular surveillance using standardized methodologies such as the Global-PPS could help hospitals and policymakers monitor prescribing trends, identify stewardship priorities, and evaluate future interventions over time. While this study does not assess the effectiveness of specific AMS interventions, it provides a benchmark for future research and stewardship activities in Nepal. The findings may also offer useful insights for other low-and middle-income countries facing similar challenges in antimicrobial use surveillance, diagnostic capacity, and AMS implementation.

## Figures and Tables

**Table 1:** Overall antimicrobial use prevalence by ward type in Nepal hospital, Global-PPS 2022–P2.

Antimicrobial Prevalence in the Medical Wards		
	Total Patients (N) *	Treated Patients (%) **
Neonatal Intensive Care Unit (NICU) ^3^	23	95.7
Adult Intensive Care Unit (AICU)	44	93.2
Pediatric Medical Ward (PMW) ^2^	30	86.7
Adult Surgical Ward (ASW)	138	59.4
Adult Medical Ward (AMW) ^1^	144	43.1

* Total patients (N) = number of admitted patients (inpatients). ** Treated patients (%) = 100 × (number of adults treated with at least one antimicrobial/number of admitted patients). ^1^ Note: AMW (adult medical ward) follows the Global-PPS ward classification protocol in use since September 2019, under which AMW denotes the general/mixed adult medical ward only; specialized adult medical wards (cardiology, CAR; dermatology—burns, DB; geriatrics, GER; infectious disease, ID; isolation, IS; long-term care, LTC; neurology, NEU; gynecology—obstetrics, OBG; psychiatry, PSY; rehabilitation, REH; nephrology, REN) are classified separately under this protocol. None of the participating hospitals had patients admitted to any of these specialized ward categories on the survey day, except gynecology—obstetrics (OBG; N = 38) and psychiatry (PSY; N = 10), which is why no separate obstetric/gynecology ward appears in this table. The emergency department is not a recognized inpatient ward category under the Global-PPS protocol, which surveys patients only after admission to an inpatient ward; emergency department attendances were therefore not included in this survey. ^2^ PMW (pediatric medical ward) similarly denotes the general pediatric medical ward; the specialized pediatric ward categories recognized under the Global-PPS protocol (hemato-oncology pediatric medical ward, HO-PMW; T-PMW; pediatric surgical ward, PSW; pediatric intensive care, PIC) had no patients admitted at the participating hospitals during the survey. ^3^ The neonatal ward category comprises the neonatal intensive care unit (NICU) and the neonatal well-baby ward (NWW); no patients were recorded under the neonatal medical ward (NMW) subcategory during this survey.

**Table 2:** Type of infection, prophylaxis, and antimicrobial prescription patterns in Nepal hospital, Global-PPS 2022–P2.

Category	N	%
**Type of infection ᵃ**		
Community-acquired infections (CAIs)—Empirical therapy	180	92.3
Community-acquired infections (CAIs)—Targeted therapy	15	7.7
Hospital-acquired infections (HAIs)—Empirical therapy	29	100
Hospital-acquired infections (HAIs)—Targeted therapy	0	0
**Type of prophylaxis ^b^**		
Surgical antimicrobial prophylaxis (SAP)—single day	8	8.5
Surgical antimicrobial prophylaxis (SAP)—extended beyond 24 h	86	91.5
Medical antimicrobial prophylaxis (MAP)	59	38.6
**Antimicrobial prescription patterns**		
Parenteral therapy *	180	86.1
Multiple antibiotic patients **	102	48.8
Multiple antibiotics per diagnosis ***	92	41.6

Abbreviations: CAI = community-acquired infection; HAI = hospital-acquired infection; SAP = surgical antimicrobial prophylaxis; MAP = medical antimicrobial prophylaxis. ^a^ Percentages based on 224 therapeutic antimicrobial prescriptions. ^b^ Percentages based on 153 prophylactic antimicrobial prescriptions. * Based on 209 patients receiving therapeutic antimicrobial treatment (systemic antibacterials, ATC J01, only; neonatal wards NMW and NICU excluded). ** Multiple antibiotic patients = received >1 antibiotic (J01) at patient level. *** Multiple antibiotics per diagnosis = received >1 antibiotic (J01) for a single identified reason to treat (diagnosis code) at patient level.

**Table 3:** Class of antimicrobial prescribed in Nepal hospitals, Global-PPS 2022–P2.

Antimicrobial Class	N	%
Systemic antibacterials	231	60.9
Nitroimidazole derivatives	7	1.8
Antifungal agents	2	0.5
Anti-tuberculosis drugs (ATBs)	1	0.3
Intestinal anti-infectives	1	0.3

Note: Percentages based on 379 admitted patients.

**Table 4:** Common diagnoses and their most frequently reported indications for antimicrobial use in Nepal hospitals, Global-PPS 2022–P2.

Indication	Prevalence (%)	Antibiotic Prescribed	Patient Prescribed (%)
Hospital-acquired pneumonia	20.5	Meropenem (J01DH02)	21.5
		Ceftriaxone (J01DD04)	19.7
		Azithromycin (J01FA10)	17
Lower UTI (treatment)	13.1	Levofloxacin (J01MA12)	19.5
		Ceftriaxone (J01DD04)	17.1
		Piperacillin/tazobactam (J01CR05)	17.5
UTI surgical prophylaxis	9.8	Imipenem/cilastatin (J01DH51)	69
		Piperacillin/tazobactam (J01CR05)	37.5
UTI medical prophylaxis		Doxycycline (J01AA02)	100
Bronchitis	4.1	Not specified	N/A
Intra-abdominal sepsis	4.1	Not specified	N/A

ATC codes are presented according to the Anatomical Therapeutic Chemical (ATC) classification. Prescription rate (%) refers to the proportion of antimicrobial prescriptions within each clinical indication. N/A = not applicable.

**Table 5:** Antimicrobial prescribing quality indicators in participating hospital sites by ward.

Indicators	Adults N (%)	Neonatal N (%)	Overall N (%)
Medical wards			
Indication documented in notes	50(56.8)	23 (74.2)	73 (61.3)
Guidelines compliant	18 (54.5)	17 (70.8)	35 (61.4)
Guidelines not available	42 (47.7)	2 (6.5)	44 (37.0)
Stop/review date	50 (56.8)	13 (41.9)	63 (52.9)
Surgical wards			
Indication documented in notes	112 (87.5)	0 (0)	112 (87.5)
Guidelines compliant	61 (73.5)	0 (0)	61 (73.5)
Guidelines not available	4 (3.1)	0 (0)	4 (3.1)
Stop/review dates	59 (46.1)	0 (0)	59 (46.1)
ICUs			
Indication documented in notes	61 (81.3)	49 (100.0)	110 (88.7)
Guidelines compliant	7 (28.0)	0.0	7 (28.0)
Guidelines not available	32 (42.7)	49 (100.0)	81 (65.3)
Stop/review dates	56 (74.7)	49 (100.0)	105 (84.7)

## Data Availability

The original data presented in this study are included in the article.

## Abbreviations

The following abbreviations are used in this manuscript:

Global-PPS	Global Point Prevalence Survey
AMS	Antimicrobial Stewardship
LMICs	Low- and Middle-Income Countries
ICU	Intensive Care Unit
HAI	Hospital-Acquired Infection
CAI	Community-Acquired Infection
ATB	Anti-tuberculosis drugs
AMR	Antimicrobial Resistance
UTI	Urinary Tract Infection
PPS	Point Prevalence Survey
AMC	Antimicrobial Consumption
WHO	World Health Organization
ATC	Anatomical Therapeutic Chemical (classification system)
AWaRe	Access, Watch, Reserve (WHO antibiotic classification)
GLASS	Global Antimicrobial Resistance and Use Surveillance System
NAP-AMR	National Action Plan on Antimicrobial Resistance
MoHP	Ministry of Health and Population (Nepal)
NICU	Neonatal Intensive Care Unit
SAP	Surgical Antimicrobial Prophylaxis
MAP	Medical Antimicrobial Prophylaxis
